# Primary Refractory Catastrophic Antiphospholipid Syndrome Masquerading as Buerger’s Disease

**DOI:** 10.7759/cureus.14350

**Published:** 2021-04-07

**Authors:** Toufic Tannous, Claudia Rosso, Jenna Iannuccilli, Karim Tannous, Matthew Keating

**Affiliations:** 1 Internal Medicine, Roger Williams Medical Center/Boston University, Providence, USA; 2 School of Medicine, University of California - Irvine, Irvine, USA; 3 Internal Medicine, Lifespan Physician Group, Providence, USA; 4 School of Medicine, University of Balamand, Tripoli, LBN; 5 Division of Hematology and Oncology, University of California - Irvine, Irvine, USA

**Keywords:** catastrophic antiphospholipid syndrome, eculizumab, thromboangiitis obliterans

## Abstract

Catastrophic antiphospholipid syndrome (CAPS) is a lethal disease with up to 30% mortality rate. It can occur as a primary disease or secondary to an underlying autoimmune disease. Current treatment focuses on disease control with anticoagulation and steroids. Plasma exchange and intravenous immunoglobulin (IVIG) have shown some benefit when added. Monoclonal drugs such as rituximab have shown some benefit in refractory cases, and eculizumab, a drug approved for use in atypical hemolytic uremic syndrome, has demonstrated disease control in a few case reports. We describe a unique case of primary refractory CAPS with an unusual presentation that was treated with five lines of therapy before disease control was established.

## Introduction

Antiphospholipid syndrome (APS) is an acquired condition that can occur as primary APS with no known underlying condition or secondary to an underlying autoimmune disease such as systemic lupus erythematosus (SLE) [1]. It is an autoimmune disorder that leads to either venous or (less commonly) arterial thrombosis and is associated with loss of pregnancy. Multiple antiphospholipid antibodies have been linked to the disease and are considered part of its diagnostic criteria, including anticardiolipin immunoglobulin G (IgG) and immunoglobulin M (IgM), lupus anticoagulant, and anti-beta-2 glycoproteins IgG and IgM [2]. Only one antibody needs to come back positive in the setting of thrombosis to diagnose APS. The disease has no definitive day-to-day treatment aside from lifelong anticoagulation (AC) to prevent further thrombotic events [3]. Multiplicity of antiphospholipid antibodies impacts the choice of AC as evidenced by the Trial on Rivaroxaban in AntiPhospholipid Syndrome (TRAPS), which indicated superior thrombotic outcomes for Coumadin versus rivaroxaban in triple-positive APS patients [4].

Catastrophic APS (CAPS) is a severe variant of the disease considered to be very rare and characterized by small vessel occlusions and thrombosis with multi-organ failure [5]. This is a life-threatening condition with a mortality of over 30%, and nearly 50% when associated with lupus [6]. Currently, an international CAPS registry exists where more than 500 cases have been collected and reviewed by the European Forum on Antiphospholipid Antibodies [7]. The pathophysiology remains somewhat unclear; however, the process involves the APS antibodies igniting a thrombotic and cytokine storm, resulting in a systemic inflammatory response syndrome (SIRS) [8]. CAPS is believed to be triggered by infections, malignancy, SLE, or pregnancy in two-thirds of the cases. Those that have no obvious underlying trigger are considered “primary” [9]. Diagnosis is based on the preliminary criteria for classification of CAPS. A “definitive” CAPS diagnosis requires “evidence of involvement of three or more organ systems; development of manifestations simultaneously or within less than a week; confirmation by histopathology of small vessel occlusion in at least one organ or tissue; lab confirmation of the presence of the APS antibodies.” A “probable” CAPS diagnosis includes at least three complete criteria with an incomplete fourth. These diagnostic criteria have been described in detail by Cervera et al. [5]. It remains very important to point out that repeat testing of the APS antibodies is required 12 weeks after the first test to confirm a CAPS diagnosis.

AC is considered the cornerstone of treatment in CAPS [10]. Steroids are recommended for the purpose of blunting the SIRS [7]. Plasma exchange (usually used in thrombotic thrombocytopenic purpura [TTP]) in addition to steroids and AC has shown disease control [11]. Similar to plasma exchange, intravenous immunoglobulin (IVIG) treatment given alongside steroids and AC has shown some benefit [7]. Cyclophosphamide is another option used in SLE patients [12]. Rituximab, an anti-CD20 monoclonal antibody, has also been used in CAPS with underlying malignancy or in refractory cases [13]. Finally, eculizumab, a monoclonal antibody used to treat atypical hemolytic uremic syndrome (HUS) that blocks C5 complement activation, has also been used off-label for refractory CAPS cases when triple therapy failure has occurred. So far, its use is mostly based on results from case reports and animal models [14,15].

## Case presentation

A 49-year-old male presented to the rheumatology clinic with mild discoloration involving his fingers and toes for a few days. His past medical history was significant for alcohol abuse and hypertension. His past surgical history was significant for a cholecystectomy and hernia repair. His past family history was non-contributory, and he denied recreational drug use. Rheumatologic workup at that point included antinuclear antibody (ANA), rheumatoid factor (RF), serum antibodies such as anti-SCL-70, anti-RNA polymerase, anti-centromere, and protein C and S, prothrombin gene mutation, factor V Leiden mutation, and hepatitis C panel with cryoglobulin assay. All results came back within normal limits. Due to his smoking history, he was presumed to have thromboangiitis obliterans ([TAO] Buerger's disease) and was prescribed a calcium channel blocker (CCB) in place of his beta blocker and was advised to stop smoking.

He developed progressive, severe pain and ulcerations to his fingers and toes over the following week which prompted hospitalization. He denied fever, chills, chest pain, shortness of breath, blurry vision, headache, or any muscle weakness. Vital signs were within normal limits, except for a mild tachycardia of 105 bpm due to the pain. Physical examination was benign aside from bluish discoloration of the distal part of the fingers and toes with multiple minute ulcerations. Radial and ulnar pulses were intact.

Laboratory workup on admission is detailed in Table 1.

**Table 1:** Laboratory workup on admission

Laboratory test	Reference Range	Patient’s Results
Hemoglobin	14-18 gm/dL	11.4 gm/dL
Platelet count	150-450x10^3/^uL	77x10^3^/uL
Erythrocyte sedimentation rate	0-18 mm/hr	91 mm/hr
Reticulocyte count percentage	0.5-2%	0.7%
Haptoglobin level	36-195 mg/dL	313 mg/dL
Creatinine	0.5-1.1 mg/dL	1.1 mg/dL
Lactic acid level	4.5-19.8 mg/dL	13.3 mg/dL
Total bilirubin	0.2-1 mg/dL	0.3 mg/dL
Lactate dehydrogenase level	140-271 U/L	199 U/L
C-reactive protein	0-7.3 mg/dl	177.44 mg/dL
Aspartate transaminase	10-42 U/L	49 U/L
Alanine transaminase	10-60 U/L	45 U/L
Ferritin level	24-336 ng/mL	94.7 ng/mL
Prothrombin time	9-11 seconds	10.2 seconds
International normalized ratio	0.9-1.1	1
Partial thromboplastin time	22-32 seconds	28 seconds
Iron level	50-170 mcg/mL	12 mcg/mL
Transferrin iron binding capacity	250-450 mcg/dL	283 mcg/dL
Fibrinogen	206-408 mg/dL	492 mg/dL
D-dimer	0.19-0.52 mg/L FEU	7.64 mg/L FEU

He was started on morphine for pain control and nifedipine 10 mg three times a day intravenously for presumed TAO crisis and admitted to the hospital for further workup and pain management.

His platelet (Plt) count and hemoglobin (Hb) level on admission were noticeably lower compared to the workup done a few days prior at the rheumatology clinic (Hb of 13 gm/dL and Plt count of 266x10^3^/uL). In addition, his creatinine (Cr) level kept worsening, with an associated decrease in urine output. This prompted further investigation for anemia and thrombocytopenia in the setting of acute kidney injury. Differentials at that point included HUS, TTP, sepsis, or any underlying neoplastic process. Further workup included a peripheral blood smear, protein electrophoresis and serum immunofixation, cold agglutinins, ANA screen, proteinase-3, myeloperoxidase and glomerular basement membrane antibodies, double-stranded DNA and smith antibodies, anti-U1-RNP and histone IgG antibodies, APS antibodies including beta-2 glycoprotein IgG and IgM, anti-cardiolipin IgA, and cardiolipin IgG and IgM antibodies, partial thromboplastin time (PTT)-lupus anticoagulant screen, complement levels (C3 and C4), hepatitis, HIV, parvovirus, and syphilis serologies, stool testing for Shiga toxin, ADAMTS13 (a disintegrin and metalloproteinase with a thrombospondin type 1 motif, member 13) assay, platelet factor-4 antibodies, blood cultures, and computed tomography (CT) of the chest/abdomen/pelvis. All results came back within normal limits except for the PTT-lupus anticoagulant screen that yielded a result of 44 seconds (normal levels < 40 seconds) with a positive hexagonal phase confirmation. No antibiotics were on board, and there were no other obvious causes of drug-induced thrombocytopenia. Although prophylactic low molecular weight heparin (LMWH) was initiated upon admission, his thrombocytopenia predated this medication and the platelet factor-4 antibodies were negative, arguing against heparin-induced thrombocytopenia. In the absence of neurologic symptoms with normal ADAMTS13 levels and no diarrhea or Shiga toxin, our differential shifted away from the TTP-HUS spectrum and we started to entertain the idea of CAPS. He was switched to therapeutic LMWH and started on prednisone. Results of a punch biopsy of the skin from one of the affected fingers revealed fibrin thrombi occluding the small arteries in the deep dermis/subcutis with focal lymphocytic inflammation. These findings were consistent with a thrombotic microangiopathy (TMA) and established a provisional clinical diagnosis of CAPS.

**Figure 1:**
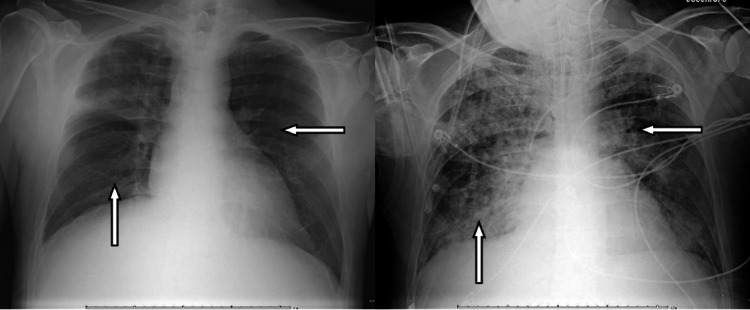
Changes seen in the chest X-ray between day 1 and day 5. The left image shows clear lungs, whereas the right image shows diffuse interstitial and alveolar infiltrates.

Due to his worsening kidney injury, anemia, and thrombocytopenia (Cr of 7.8 mg/dL, Hb level of 5.3 gm/dL, and Plt count of 23x10^3^/uL), LMWH was stopped, nephrology was consulted, and the patient was started on hemodialysis as well as plasma exchange on day 4 of hospitalization for suspicion of CAPS and a kidney biopsy was planned. One day later, he became acutely short of breath, and a chest X-ray (Figure 1) showed diffuse multilobar consolidations. Due to his swift clinical deterioration and acute respiratory failure, he was intubated and sent to the ICU for further management. A bronchoscopy was performed and showed diffuse alveolar hemorrhage (DAH). Results of the kidney biopsy showed TMA involving glomeruli and arterioles (associated with lupus anticoagulant).

**Figure 2:**
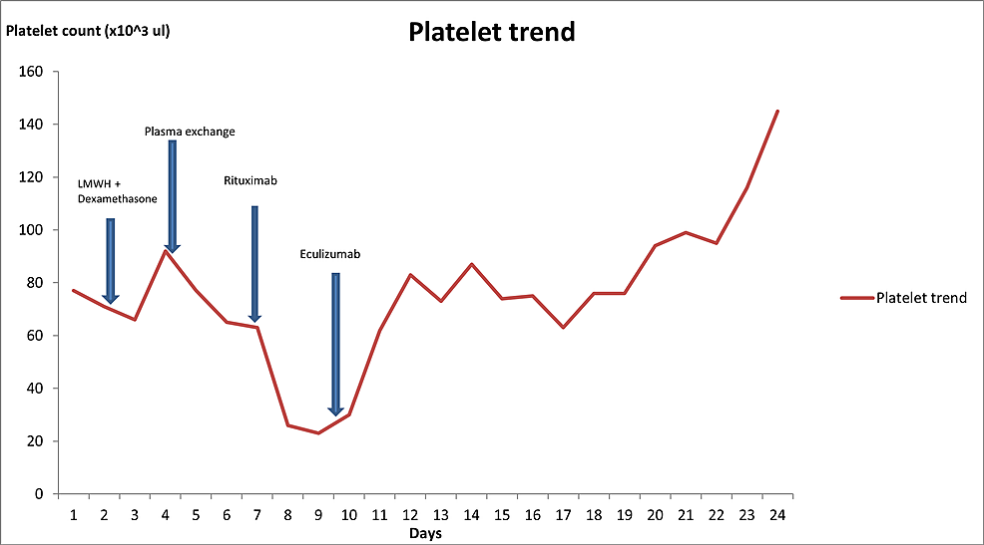
Platelet count trend during hospitalization with the addition of every treatment (note: no platelet transfusions were given).

**Figure 3:**
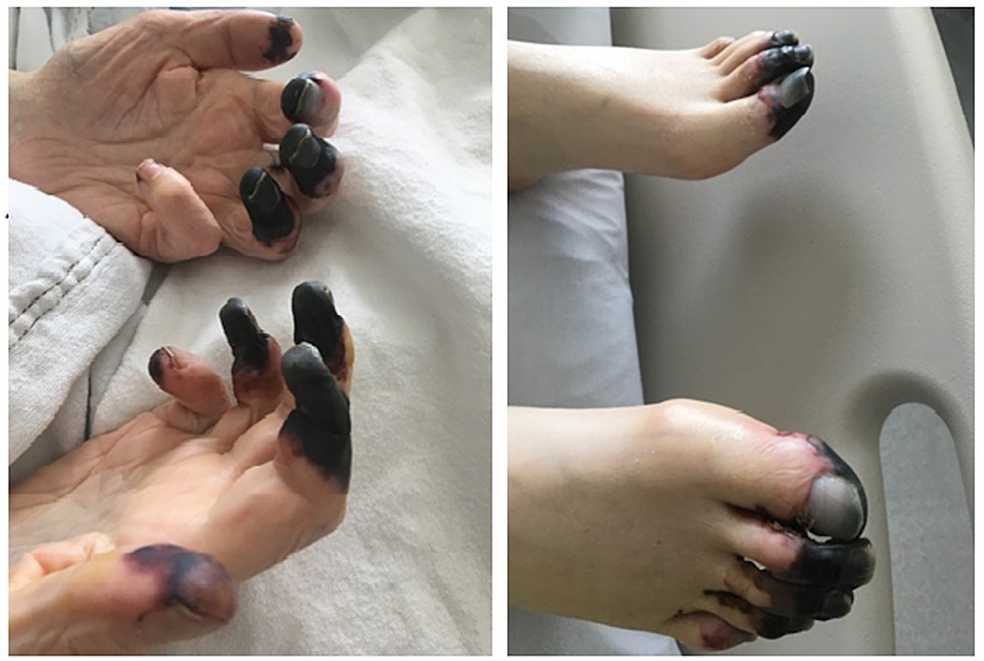
Images of our patient’s distal necrosis affecting his fingers and toes, obtained with permission from the patient.

The culmination of several clinical and laboratory findings led to a diagnosis of refractory CAPS, which included unresponsiveness to steroids and plasma exchange, abnormal skin, renal biopsy results, and a positive lupus anticoagulant test. The plan moving forward was to start rituximab 500 mg and eculizumab 900 mg once every week for a total of four weeks. He was initially started on rituximab with no significant improvement. He was provided with the prerequisite meningococcal and streptococcal vaccinations prior to receiving eculizumab. One week after the addition of eculizumab, his blood counts and kidney function started to improve (Plt count of 76x10^3^/uL and Cr of 1.8 mg/dL). AC was resumed, and weekly rituximab and eculizumab treatments were continued. Figure 2 depicts the progression of the platelet trend after the addition of each treatment. Two weeks later, the patient was extubated and dialysis was stopped after his kidney function improved. Eventually his fingers and toes required distal amputations as they had become necrotic during his hospital stay (Figure 3). After finishing his treatment, he was discharged on lifelong Coumadin and instructed to follow up in the outpatient setting. He had a repeat lupus anticoagulant test six weeks later while on Coumadin, which came back negative.

## Discussion

The earliest report of CAPS was published in 1984 by Dosekun et al. They described the case of a patient with SLE who presented with multi-organ failure, several thrombotic events, and lab finding of an in vitro urokinase-induced fibrinolysis process in his serum. Treatment with Ancrod, a defibrinogenating agent derived from the Malaysian pit viper, resulted in recovery [16]. Soon after, multiple cases of CAPS were reported, leading to the formation of a CAPS registry with more than 500 cases documented thus far [7]. Although rare, cases reporting the successful treatment of refractory CAPS with eculizumab are increasing in number; however, there remain no randomized controlled trials to prove its efficacy. The pathophysiology of CAPS and other TMAs involves perturbation of terminal complement activation; therefore, it follows that the terminal complement inhibitor eculizumab would yield promising results. Most existing case reports and case series describe patients with a background of SLE [15,17] or previously diagnosed APS [17], but very few reports have described primary refractory CAPS treated with eculizumab [18]. The presentations in these cases range from pregnancy-related complications such as proteinuria, hypertension, and thrombocytopenia, to cerebrovascular ischemic strokes, fulminant renal failure, pulmonary emboli, and limb ischemia. As mentioned earlier, most of these patients were diagnosed with autoimmune diseases, and the diagnosis of CAPS more readily follows in the presence of these known secondary causes. This allows quick treatment decision-making and escalation of care in an attempt to reduce mortality and preserve organ function. Another interesting observation is that many of the patients had a history of idiopathic thrombotic events, most commonly pulmonary embolism. The importance of these thrombotic events is that they serve as a prelude to the more dire manifestations that may come later on. Possibly, when given enough consideration, the initial thrombotic events can lead to a hypercoagulable workup to identify APS before CAPS and thereby help prevent the more dire manifestations from occurring.

The experience with off-label use of eculizumab in these refractory cases has shown disease reversal based on published case reports as well as a case series of nine patients by Kello et al. [17]. The success of eculizumab after failure of triple therapy brings hope that control of the disease is still possible. More cases reporting similar success may prompt more studies to be conducted on the drug, though admittedly a randomized trial in CAPS patients may not be practical.

Our case serves to add to the growing evidence regarding refractory CAPS and treatment with eculizumab. The initial presentation with discoloration of the fingers and toes was misleading. The focus in the rheumatology clinic was to rule out major rheumatologic diseases as secondary causes (SLE, rheumatoid arthritis, scleroderma), but since workup and history were unrevealing, it made sense with his smoking history that a diagnosis of TAO was made. Although he was slightly older than the average age at which TAO is diagnosed, the patient did fit the stereotypical presentation: male with more than 20-year smoking history [19]. However, we did not have a biopsy to confirm yet, and empiric treatment was started with a CCB. At this point when his condition worsened, there was a swift decline in his overall clinical status with multi-organ failure, which was perplexing at the time. This was an unusual presentation for TAO. This led us to consider other conditions that could potentially present as such, with a broad differential that even included thymoma-associated multi-organ autoimmunity (TAMA) diseases. TAMA is partially characterized by cutaneous manifestations, such as necrosis, variable success with systemic steroids, and multi-organ failure. It is often associated with paraneoplastic or autoimmune diseases such as myasthenia gravis [20]. However, our patient never carried any autoimmune disease diagnosis, and there were no apparent inciting stressors identified that may have triggered his condition. Eventually, the diagnosis was made after laboratory and pathological confirmation, but two issues remained. He was not responding to steroids, plasma exchange, or AC, and we had to stop the AC due to DAH. At this point, it became apparent that our patient had a primary refractory case of CAPS. Based on the literature mentioned earlier, our treatment options were slim. We started rituximab initially based on the benefit it had shown in a recent review of refractory patients from the CAPS registry [13]; however, no significant response was obtained in our patient. Our only option left was eculizumab. Within a week, we started seeing improvement in the overall clinical and laboratory status.

One word of caution: our case does not qualify for the “definitive CAPS” diagnosis but instead fits “probable CAPS” as defined earlier. The reason for the discrepancy is that the lupus anticoagulant repeated six weeks after the initial diagnosis came back negative. However, this might be a false-negative, as current recommendations advise re-checking the APS antibody testing 12 weeks after the initial diagnosis. The period of 12 weeks was chosen due to the probable effect of plasma exchange that might linger before the antibodies reappear in the serum [2].

## Conclusions

CAPS can present in a myriad of ways; high suspicion and immediate recognition help in preventing treatment delays. The use of eculizumab in cases of primary and secondary refractory CAPS has demonstrated disease control and even reversal. This is mostly based on anecdotal evidence from case reports and case series. More studies are needed to justify its use.
